# CRAF mutations in lung cancer can be oncogenic and predict sensitivity to combined type II RAF and MEK inhibition

**DOI:** 10.1038/s41388-019-0866-7

**Published:** 2019-07-08

**Authors:** Amir Noeparast, Philippe Giron, Alfiah Noor, Rajendra Bahadur Shahi, Sylvia De Brakeleer, Carolien Eggermont, Hugo Vandenplas, Bram Boeckx, Diether Lambrechts, Jacques De Grève, Erik Teugels

**Affiliations:** 1 Laboratory of Molecular Oncology and Department of Medical Oncology, Oncologisch Centrum, UZ Brussel, Vrije Universiteit Brussel, Brussels, Belgium; 2 Vesalius Research Center, VIB, 3000 Leuven, Belgium; 3 Laboratory of Translational Genetics, Department of Oncology KU Leuven, 3000 Leuven, Belgium

**Keywords:** Predictive markers, Targeted therapies

## Abstract

Two out of 41 non-small cell lung cancer patients enrolled in a clinical study were found with a somatic CRAF mutation in their tumor, namely CRAF^P261A^ and CRAF^P207S^. To our knowledge, both mutations are novel in lung cancer and CRAF^P261A^ has not been previously reported in cancer. Expression of CRAF^P261A^ in HEK293T cells and BEAS-2B lung epithelial cells led to increased ERK pathway activation in a dimer-dependent manner, accompanied with loss of CRAF phosphorylation at the negative regulatory S259 residue. Moreover, stable expression of CRAF^P261A^ in mouse embryonic fibroblasts and BEAS-2B cells led to anchorage-independent growth. Consistent with a previous report, we could not observe a gain-of-function with CRAF^P207S^. Type II but not type I RAF inhibitors suppressed the CRAF^P261A^-induced ERK pathway activity in BEAS-2B cells, and combinatorial treatment with type II RAF inhibitors and a MEK inhibitor led to a stronger ERK pathway inhibition and growth arrest. Our findings suggest that the acquisition of a CRAF^P261A^ mutation can provide oncogenic properties to cells, and that such cells are sensitive to combined MEK and type II RAF inhibitors. CRAF mutations should be diagnostically and therapeutically explored in lung and perhaps other cancers.

## Introduction

The RAF kinase family, which consists of three isoforms, ARAF, BRAF, and CRAF (RAF1), transmit signal from RAS to MEK along the RAS/RAF/MEK/ERK molecular pathway [1]. RAF kinase family members share three conserved regions (CR1-CR3) [1]. The kinase activity of CRAF is higher than ARAF but lower than BRAF [1, 2]. BRAF and CRAF germline mutations have been previously described in rasopathies [2–4]. Somatic BRAF mutations have been detected in ~8% of human tumors including non-small cell lung cancer (NSCLC) (5%) and melanoma (~50%), whereas CRAF mutations are very rarely reported in cancer [1, 3]. With the emergence of genome-wide next-generation sequencing, somatic CRAF mutations in cancer appear to occur more frequently than previously considered [4–6]. We performed whole exome sequencing of 41 available paired tumor/matched normal tissue samples derived from a prospective cohort of “strictly non-smoking” or “formerly limited smoking” NSCLC patients and detected two CRAF mutations, namely CRAF^P261A^ and CRAF^P207S^ (manuscript in preparation). To our knowledge, these CRAF mutations have never been reported in lung cancer. One of these mutations, CRAF^P261A^, is located in conserved region CR2 and has never been reported in human cancer. However, a CRAF^P261A^ germline mutation was reported in Noonan syndrome (rasopathy) and its characterization revealed that it activates the ERK pathway at higher levels compared with CRAF^WT^ [7].

Markedly, the 14-3-3 proteins can bind to the CR2 of CRAF, at the phosphorylated S259 (and with lower affinity at p-S233), thereby stabilizing the CRAF auto-inhibition state [1, 8–10]. CRAF mutations at CR2 can affect the 14-3-3-binding motif or its recognition by phosphatases, and thereby promoting CRAF kinase activation [1, 7, 11].

The other mutation, CRAF^P207S^, located at a non-conserved region between CR1 and CR2, was previously identified in a fibrosarcoma cell line and reported as incapable of activating the ERK pathway at higher levels than wild-type CRAF and its role as an oncogene remained undetermined [2].

Predicting the efficacy of RAF inhibitors in targeting mutated CRAF is still a challenge. A melanoma-derived oncogenic CRAF mutation (CRAF^R391W^), which signals as a dimer, is reported to be resistant to Vemurafenib (a type I RAF inhibitor) [12]. Of note, acquisition of mutations at S259 or adjacent residues of CRAF including P261 [13] has also been described as one of the resistance-conferring mechanisms to type I RAF inhibitor therapy in mutant BRAF^V600E^ melanomas [13]. In contrast, lung cancer-derived mutations at S259 and S257 CRAF have been shown to predict sensitivity to Sorafenib, a type II RAF and multiple kinase inhibitor [6].

In the present work, we investigated the “actionability” of these lung cancer-derived CRAF mutations with ERK pathway inhibitors (RAF and MEK inhibitors) and further determined the comparative efficacy of two classes of RAF inhibitors in targeting these mutations.

## Results and discussion

### CRAF^P261A^ but not CRAF^P207S^ increases ERK pathway activity in a dimer-dependent manner

**Fig. 1:**
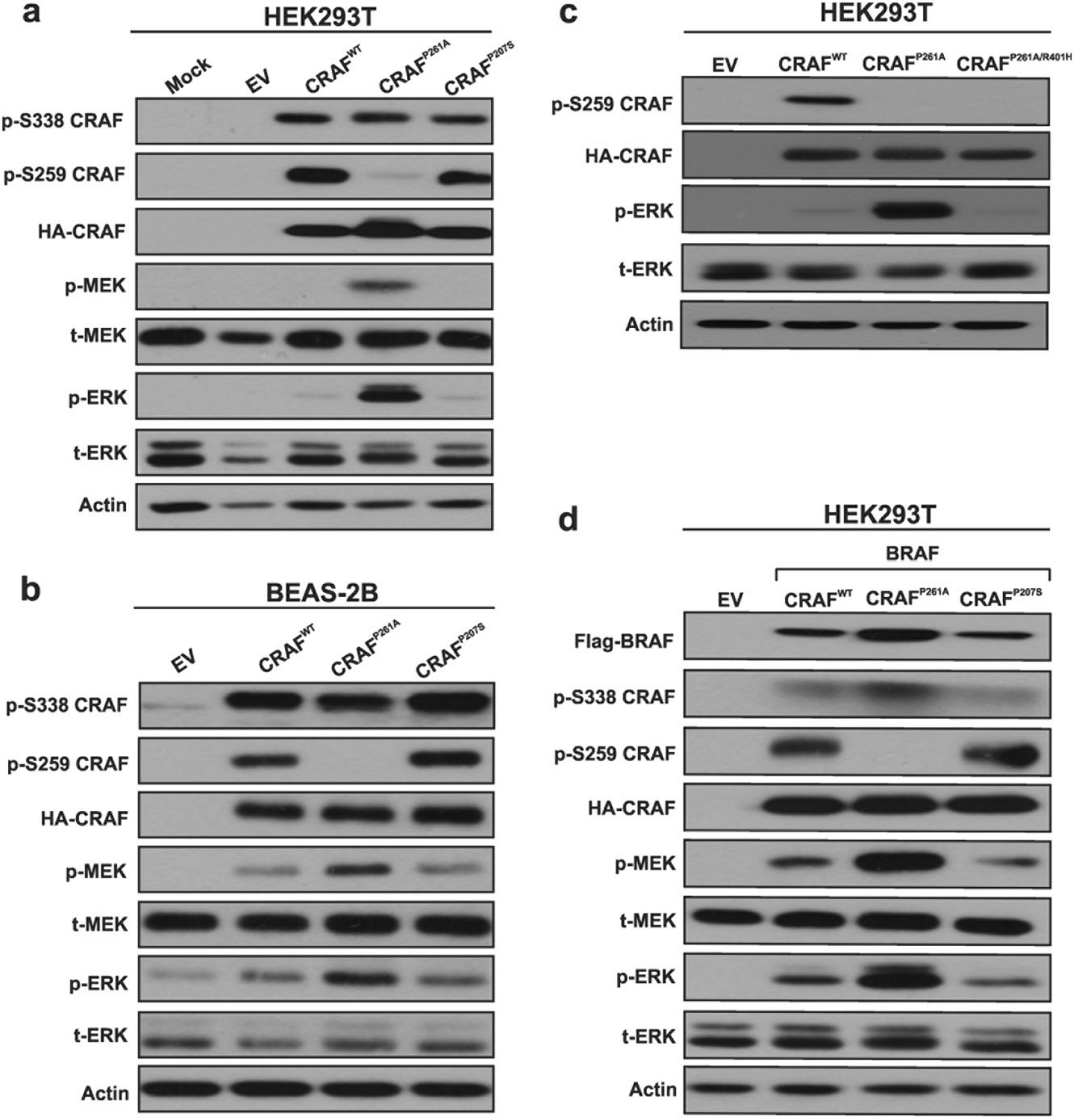
CRAF^P261A^ activates the ERK pathway in HEK293T and BEAS-2B cells, and signals as a dimer. HEK293T (**a**) or BEAS-2B cells (**b**) cells were transiently transfected with a CRAF^P261A^, CRAF^P207S^, or CRAF^WT^ expression vectors alone. The CRAF^P261A^ mutation hyperactivates the ERK pathway. The prevention of CRAF dimerization (by CRAF^R401H^) abolishes CRAF^P261A^ hyperactivation of the ERK pathway in HEK293T cells (**c**). Co-expression of CRAF^WT^, CRAF^P261A^, or CRAF^P207S^ with BRAF^WT^ (as previously described [22]) in HEK293T cells do not result in higher (or lower) ERK pathway activation compared with single transfections (**d**). Forty-eight hours post-transfection cells were lysed and subjected to western blotting analysis to detect the indicated proteins (**a**, **b**, **c**, **d**). EV stands for empty vector. Lipofectamine was added in the mock condition

To determine whether CRAF^P261A^ and CRAF^P207S^ mutations can induce ERK pathway activation at higher levels compared with the wild-type CRAF, we introduced CRAF^P261A^ and CRAF^P207S^ mutations into the wild-type CRAF coding sequence by site-directed mutagenesis and transiently expressed the mutant CRAF recombinant proteins in HEK293T and BEAS-2B cells. As shown in Fig. 1a, b, the expression of CRAF^P261A^ led to increased MEK and ERK activation in both HEK293T and BEAS-2B cellular models. The enhanced MEK and ERK activity induced by CRAF^P261A^ was less pronounced in BEAS-2B cells, which could be explained by a lesser transfection efficiency of BEAS-2B cells as opposed to HEK293T cells. It was previously reported that phosphorylation of CRAF at S338 is crucial for its activation, linking it to cancer progression [14–16], whereas phosphorylation at residue S259 (a negative regulatory site adjacent to P261) is essential for CRAF auto-inhibition [10, 17]. In all tested conditions we observed that increased ERK pathway activity induced by CRAF^P261A^ was accompanied by a clear decline in S259-CRAF phosphorylation levels (Fig. 1a, b). In contrast, no marked increase in S338-CRAF phosphorylation levels was observed (Fig. 1a, b).

RAF targeting studies including a recent study by us have shown that monomer vs. dimer signaling of BRAF can determine its mode of response to RAF inhibition [5, 18–31]. However, whether CRAF^P261A^ functions as a monomer or a dimer, and whether it predicts sensitivity to the ERK pathway inhibitors were yet to be uncovered. Therefore, we introduced the well-studied dimerization-disrupting R401H mutation in CRAF^P261A^ [12, 25, 31, 32]. We observed that CRAF^P261A/R401H^ could no longer induce ERK pathway activation, indicating that CRAF^P261A^ signals as a dimer and not as a monomer (Fig. 1c). This observation suggests that CRAF^P261A^ still requires dimerization to allow hyperactivation of the ERK pathway, following disruption of 14-3-3 proteins binding to its N-terminal binding motif, as indicated by loss of S259 phosphorylation. Similar findings have been reported for BRAF^S365A^ (BRAF^S365^ is homologous to CRAF^S259^), which is also impaired in 14-3-3 binding to the CR2 [33].

In contrast to CRAF^P261A^, CRAF^P207S^ did not induce MEK and ERK phosphorylation at higher levels compared with CRAF^WT^ (Fig. 1a, b). We postulated that CRAF^P207S^, like kinase-impaired BRAF mutants that allosterically activate their heterodimerization partner (CRAF), might function in co-operation with wild-type BRAF. Therefore, we overexpressed CRAF recombinant proteins in HEK293T cells together with BRAF^WT^. As shown in Fig. 1d, co-expression of BRAF together with CRAF^P207S^ did not lead to increased MEK and ERK activation when compared with CRAF^WT^/BRAF co-transfectants (idem for CRAF^P261A^).

### CRAF^P261A^ transforms mouse embryonic fibroblasts and human lung epithelial cells

**Fig. 2:**
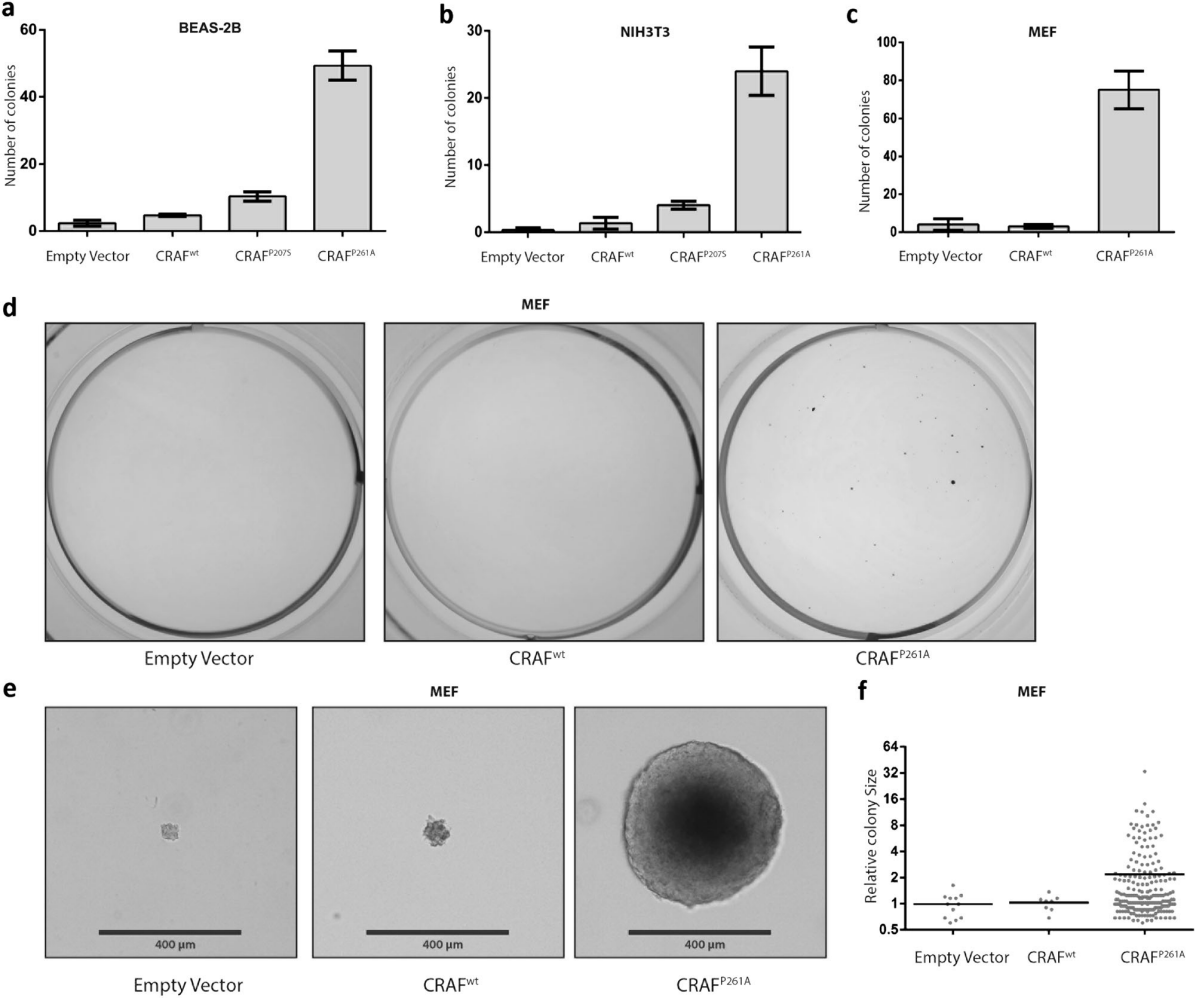
CRAF^P261A^ induces anchorage-independent growth. BEAS-2B (**a**), NIH3T3 (**b**), and MEF (**c**) cells were stably transduced with empty vector, CRAF^WT^, CRAF^P207S^ (only in BEAS-2B and NIH3T3), or CRAF^P261A^ as indicated (**a**–**c**). Total number of colonies per well were counted (representative experiment) after 16 (**c**) or 18 (**a**, **b**) days in culture. The experiments were performed with three biological repeats and were represented as a bar chart (means ± SEM). **d** Representative whole well image of the nitro blue tetrazolium chloride stained MEF colonies after 16 days in culture. **e** Representative high-magnification images of the MEF colonies prior to staining. **f** Graphical representation of the relative size of the MEF colonies as determined with OpenCFU software [55]. Dots represent individual colonies (the three biological repeats are pooled), lines represent the means

To determine whether CRAF^P207S^ and CRAF^P261A^ mutations can induce anchorage-independent growth, a hallmark of carcinogenesis, we stably transduced lung epithelial cells (BEAS-2B) and mouse embryonic fibroblasts of two different origins (NIH3T3 [34] derived from Swiss 3T3 mice and another immortalized MEF [35] derived from C57BL/6J mice, hereafter mentioned as MEF), with lentiviral vectors bearing recombinant wild-type and mutant forms of CRAF. The anchorage-independent growth was determined using a soft agar colony-formation assay, which is considered as a stringent test for malignant transformation. As shown in Fig. 2a–c, the number of colonies formed after transducing BEAS-2B, NIH3T3, and MEF cells with CRAF^P261A^ strongly increases when compared with CRAF^WT^ transductions. We observed a 10.6-fold, 18.4-fold, and 25-fold increase in colony formation for respectively BEAS-2B, NIH3T3 (the least pronounced) and MEF stably transduced cells. Comparable results were not obtained when transducing cells with CRAF^P207S^ (Fig. 2a, b). Indeed, CRAF^P207S^ exhibited only a meagre transforming activity (Fig. 2a, b), which correlates with the observed lack of ERK pathway activation (Fig. 1a, b).

In addition, CRAF^P261A^ not only generates more colonies but also clearly induces the formation of larger colonies (Fig. 2d–f, Supplementary Fig. S1d and S1e) compared with CRAF^WT^. Interestingly, the capacity of CRAF^P261A^ to form colonies in BEAS-2B was relatively high, as it was only four fold less than for the well-characterized BRAF^V600E^, which has been reported to rank among the highest ERK pathway activating BRAF mutants [22, 23, 36] (Supplementary Fig. S1a and S1e). We also tested CRAF^S259A^ as a control, and consistent with two previous reports [6, 37] we observed that this variant induces increased anchorage-independent growth in lung epithelial and MEFs (Supplementary Fig. S1).

### CRAF^P261A^-induced ERK pathway activation is suppressed by type II inhibitors, but paradoxically increased by type I inhibitors

Several studies have suggested that ERK pathway activating cancer-derived CRAF mutations are oncogenic, but their response to RAF inhibitors is still uncertain [4, 6, 13]. In this study, we first tested three RAF inhibitors (Dabrafenib, LY3009120, and AZ628) at the clinically relevant dose of 1 µM by measuring ERK pathway activity in BEAS-2B cells transiently expressing CRAF recombinant proteins. Dabrafenib is a type I RAF inhibitor (recently characterized as type 1.5 inhibitor [27]), which stabilizes the drug-bound RAF molecule in a DFG-in conformation [27]. AZ628 and LY3009120 are both type II RAF inhibitors and stabilize RAF in the DFG-out conformation [23, 26, 29]. Dabrafenib is Food and Drug Administration-approved for the treatment of V600E/K BRAF melanoma and has an affinity for CRAF as well [22, 38]; LY3009120 has been recently tested clinicaly (phase I study) in mutant BRAF and KRAS cancers (NCT02014116), and AZ628 is an experimental RAF inhibitor.

**Fig. 3:**
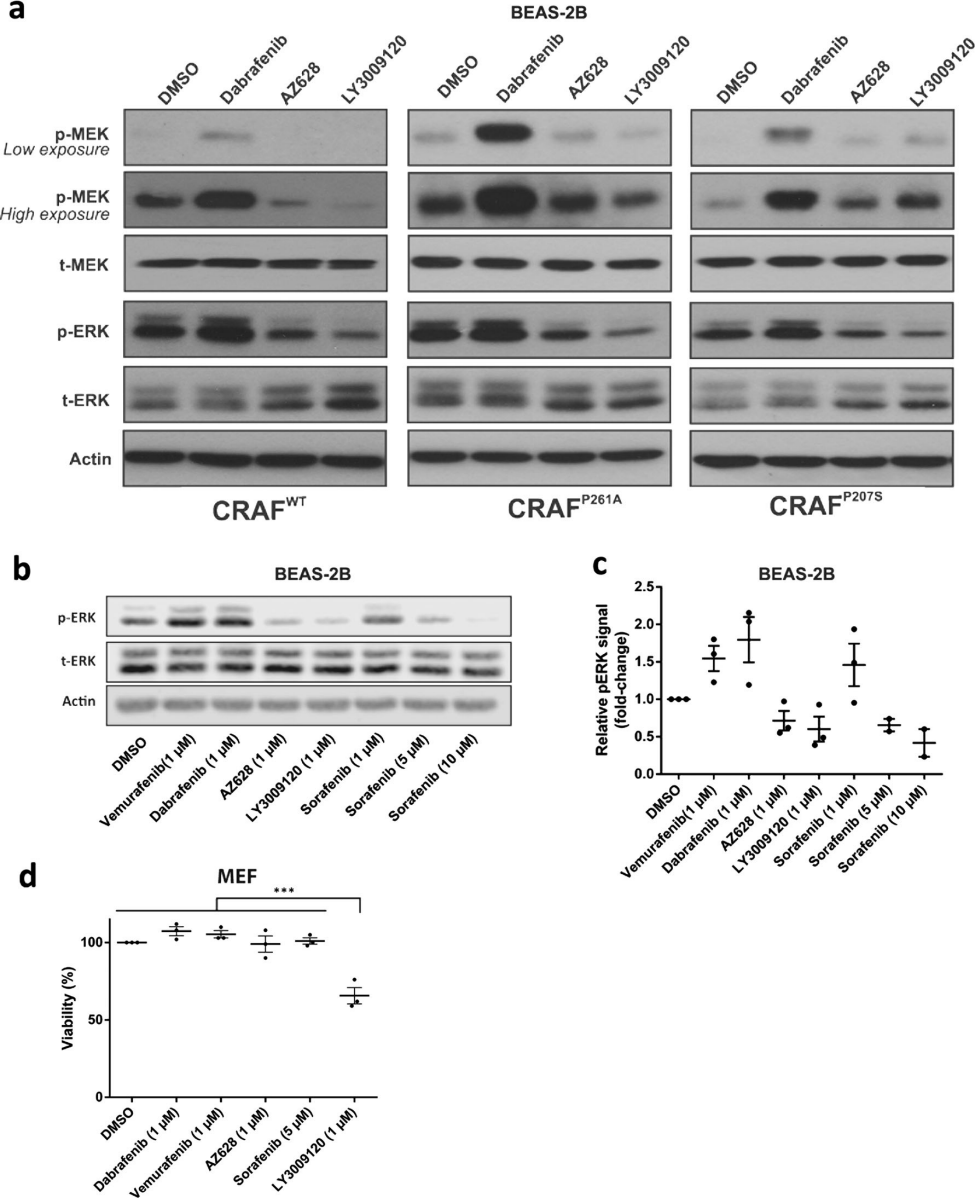
CRAF^P261A^-induced ERK pathway activation is suppressed by type II RAF inhibitors. **a** BEAS-2B cells were transiently transfected with different CRAF expression vectors (wild type or mutant). Forty-eight hours post transfection, cells were treated for 2 h with DMSO, Dabrafenib (1 µM), or AZ628 (1 µM), or LY3009120 (1 µM), then lysed and subjected to western blotting analysis to detect the indicated proteins. **b**, **c** BEAS-2B cells were transiently transfected with CRAF^P261A^. Forty-eight hours post transfection, cells were treated for 2 h with DMSO, Vemurafenib, Dabrafenib, AZ628, LY3009120, or Sorafenib (drug concentrations are indicated). **b** Cells were lysed and subjected to western blotting analysis to detect the indicated proteins. **c** Graphical representation of the relative p-ERK signals (normalized to actin and DMSO group) based on at least two independent experiments as shown in **b**. Dots represent individual data points, lines represent the mean value ± SEM. **d** MEF cells stably expressing CRAF^P261A^ were treated with DMSO, Vemurafenib, Dabrafenib, AZ628, LY3009120, or Sorafenib (all at 1 µM but Sorafenib at 5 µM) for a duration of 72 h. Cell viability was determined using CellTiter-Glo. The dots represent the means of the independent experiments, the horizontal lines with error bars represent the mean ± SEM of three independent experiments each performed at least in triplicate. Statistical significance was indicated by *** and represents a *p*-value < 0.001

Dabrafenib treatment of CRAF^WT^ expressing BEAS-2B cells whether in the absence or presence of BRAF led to paradoxical ERK pathway activation compared with dimethyl sulfoxide (DMSO) (vehicle) treatment (Fig. 3a and Supplementary Fig. S2). As previously shown [1, 21, 25, 32, 39], when a type I RAF inhibitor (Dabrafenib) binds to one CRAF^WT^ homodimer partner, the other dimer partner is transactivated, resulting in paradoxical ERK activation. Notably, a similar phenomenon was observed upon Dabrafenib treatment of cells expressing either CRAF^P261A^ or CRAF^P207S^ (Fig. 3a and Supplementary Fig. S1). In contrast, both AZ628 and LY3009120 suppressed ERK activity in CRAF^P261A^- and CRAF^P207S^-expressing cells in the presence or absence of BRAF and did not induce ERK paradoxical activity in CRAF^WT^-expressing cells (Fig. 3a and Supplementary Fig. S2). The differential effect of type I vs. type II RAF inhibition can be explained by our observation that CRAF^P261A^ relies on dimerization for downstream signaling. Indeed, the abolishment of ERK pathway signaling observed when CRAF^P261A^ mutant proteins were rendered unable to dimerize suggests that only inhibitors that concomitantly block the kinase activity of both RAF dimer partners (=type II inhibitors) can suppress CRAF^P261A^ -induced ERK pathway activation.

Interestingly, in CRAF^P207S^ mono-transfectants, both AZ628 and LY3009120 induced a slight increase in p-MEK levels, which was not consistent with the observed decline in corresponding p-ERK levels (Fig. 3a). This phenomenon was not observed in the presence of BRAF^WT^ (Supplementary Fig. S2). To further gain a broader insight into the link between the mode of RAF inhibition and the efficacy in suppressing CRAF^P261A^-induced ERK activity, we also tested (Fig. 3b, c) the clinically available Type I RAF inhibitor Vemurafenib as well as the relatively weak but clinically available Type II RAF inhibitor Sorafenib [29] (multiple kinase inhibitor). As for Dabrafenib, Vemurafenib caused paradoxical ERK activation. Also, Sorafenib activated the ERK pathway rather than inhibiting it at 1 µM, the concentration used for the other inhibitors. However, one should notice that 1 µM is a relatively low concentration for Sorafenib as the maximal plasma concentration of Sorafenib in patients reaches 10 to 21 µM [40–42]. Previous reports confirm that Sorafenib causes ERK paradoxical activation at low doses [43–45]. However, at higher doses (5 µM and 10 µM), Sorafenib caused ERK inhibition (Fig. 3b). Overall, LY3009120 exerted the strongest ERK pathway inhibition (Fig. 3a, b and Supplementary Fig. S2). These results were consistent with our observations regarding the growth inhibitory effect of the tested RAF inhibitors in MEFs transduced with CRAF^P261A^ (Fig. 3d).

### CRAF^P261A^ predicts sensitivity to the combination of LY3009120 and Trametinib

**Fig. 4:**
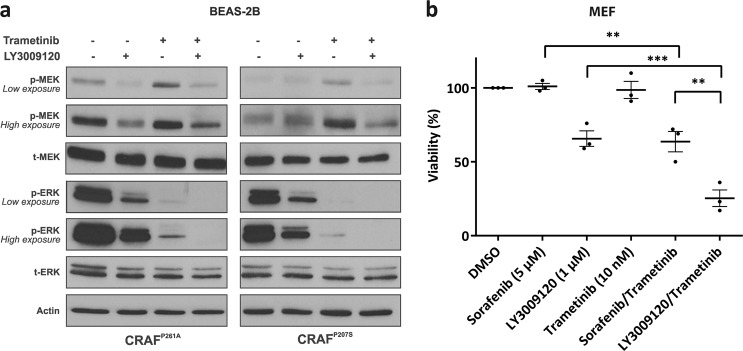
CRAF^P261A^ predicts sensitivity to combination of LY3009120 and Trametinib. **a** BEAS-2B cells were transiently transfected with different CRAF expression vectors (wild type or mutant). Forty-eight hours post transfection, cells were treated for 2 h with DMSO, LY3009120 (1 µM), and/or Trametinib (25 nM), then lysed and subjected to western blotting analysis for the indicated proteins. **b** MEF cells stably expressing CRAF^P261A^ were treated with DMSO, Sorafenib, LY3009120, Trametinib, and the combinations of Sorafenib (5 µM) or LY3009120 (1 µM) with Trametinib (10 nM), for a duration of 72 h. Cell viability was determined using CellTiter-Glo. The dots represent the means of the independent experiments, the horizontal lines with error bars represent the mean ± SEM of three independent experiments each performed at least in triplicate. Statistical significance was indicated by ** and ***, which represent *p*-values < 0.01 and 0.001, respectively

Combined MEK and BRAF targeting has shown superior efficacy in BRAF mutant cancers [18, 22, 23, 38, 39, 46–49]. We investigated whether similar effects are observed in the mutant CRAF context. MEK inhibitors are known to be ineffective or poorly effective in cells where ERK pathway activation is CRAF-mediated such as in mutant KRAS cells [50–52]. However, the MEK inhibitor Trametinib is known to be more efficient compared with several other MEK inhibitors in such cells [50]. As LY3009120 showed the highest ERK inhibitory effect among the RAF inhibitors we tested, we further studied the effect of Trametinib at the clinically relevant dose of 25 nM as a single agent and in combination with LY3009120. Trametinib single agent treatment of lung epithelial BEAS-2B cells expressing CRAF mutants alone and together with BRAF^WT^ led to increased MEK phosphorylation (Fig. 4a and Supplementary Fig. S3). However, increased MEK phosphorylation was not followed by increased ERK phosphorylation but by strong ERK inhibition. Other groups and we have previously described this phenomenon upon MEK inhibition in cells with CRAF-mediated ERK pathway activity [23, 50–53]. The ERK inhibitory effect of Trametinib alone was even stronger than for LY3009120 alone. Combination of LY3009120 and Trametinib led to an enhanced ERK inhibitory effect compared with single agent treatments, whether in the presence or absence of BRAF (Fig. 4a and Supplementary Fig. S3). Notably, the presence of LY3009120 decreased the Trametinib-induced MEK phosphorylation (Fig. 4a).

Finally, we tested the growth inhibitory effect of Trametinib (10 nM) combined with LY3009120 (1 µM) or combined with Sorafenib (5 µM) in mouse embryonic fibroblasts expressing CRAF^P261A^. In both cases, combinatorial treatments resulted in reduced cell viability compared with single agent treatments (Fig. 4b). Trametinib plus LY3009120 produced stronger growth inhibitory effects compared with Trametinib plus Sorafenib (Fig. 4b). Our findings predict sensitivity of cells with CRAF oncogene dependency to the combination of type II RAF and MEK inhibition.

In summary, two somatic CRAF mutations identified in a series of 41 NSCLC patients with a non-smoking history were examined for their ability to activate the MEK-ERK pathway and their therapeutic actionability with several pathway inhibitors. We show that one of these mutations (CRAF^P261A^) strongly activates the MEK-ERK pathway, whereas the other mutation (CRAF^P207S^) does not. The mode of RAF inhibition determines whether the ERK pathway will be suppressed or paradoxically activated in cells expressing the CRAF^P261A^ mutation. Among the type II inhibitors tested, LY3009120 resulted in strongest ERK inhibitory effect. Moreover, combined LY3009120 and Trametinib (MEK inhibitor) treatment of cells expressing CRAF^P261A^ even led to a stronger MEK and ERK inhibition. LY3009120 has shown promising preclinical efficacy in BRAF and KRAS mutant cells but the subsequent clinical trial (NCT02014116) exploring the efficacy of the inhibitor in melanoma, colon, NSCLC and pancreatic cancers led to poor pharmacodynamic responses in treated patients and discontinuation of the drug development [29, 54].

Regarding CRAF^P207S^, our results show that this mutation does not activate the ERK pathway at higher levels compared with CRAF^WT^ and does not transform the transduced cells. Further investigation is required to uncover whether CRAF^P207S^ can deregulate other pathways and induce oncogenesis in co-operation with other pro-tumorigenic events, or it is rather a passenger mutation. This study inspires discovery and characterization of CRAF mutations in lung and other cancers. Our results support the clinical exploration of the therapeutic exploitation of CRAF mutations in cancer [35].

## Supplementary information

Supplementary Information

S1

S2

S3
