# Supplementary material for: CRAF mutations in lung cancer can be oncogenic and predict sensitivity to combined type II RAF and MEK inhibition
Source: Oncogene. 2019 Jul 8;38(31):5933–41. doi: 10.1038/s41388-019-0866-7 (PMC6756226; doi:10.1038/s41388-019-0866-7)
Supplement: Supplementary file 1 — Supplementary Information [file 41388_2019_866_MOESM1_ESM.docx]

**Supplementary information: Material & Methods and** **supplementary figure legends**

**Material & Methods**

**DNA constructs**

An empty vector (puno1) and a customized HA-tagged-CRAF (puno1-hRAF1) expression vector were obtained from InvivoGen (Toulouse, France). pLenti-puro was a gift from Ie-Ming Shih^1^ (Addgene plasmid # 39481). CRAF and BRAF^2^ coding sequences were cloned into the pLenti-puro via PCR cloning using the puno1-hRAF1 and CMV14-BRAF as templates, and primers with overhangs for the Mlu1 and Xba1 restriction sites. Inserted sequences were fully sequenced.

The 3rd generation lentiviral vector packaging plasmids GAG, POL and REV were kindly provided by Prof. Brian Brown (Mount Sinai Icahn School of Medicine, NY).

**Site-directed mutagenesis**

The P207S, S259A, and P261A mutations were introduced into the wild type CRAF coding sequence by site-directed mutagenesis (GeneArt Site-Directed Mutagenesis System, Life-Technologies, A13312). Also, the CRAF^R401H^ mutation was introduced in the HA-tagged-CRAF^P261A^ construct. The whole CRAF coding sequence and the insertion sites in the generated expression vectors were fully sequenced.

**Cell culture and transfections**

NIH3T3 (ATCC, CRL-1658) and HEK293T (ATCC® CRL-3216™) cells were purchased from the ATCC. BEAS-2B^3,^^4^, and MEF^5^ cells were kindly provided by respectively Prof. Didier Cataldo^4^ and Prof. Reuven Stein^5^. HEK293T and BEAS-2B cells were cultured in Dulbecco’s Modified Eagles Medium (DMEM) (Life Technologies, 31966-047) supplemented with 10% fetal bovine serum (FBS) (Perbio Science, SV30160.03) and penicillin-streptomycin (Life Technologies, 15140-148). NIH3T3 and MEF cells were cultured in DMEM supplemented with 10% Calf Bovine Serum, Iron Fortified (ATCC, 30-2030™) and penicillin-streptomycin. Cells were passaged every three days. Transfections and co-transfections were performed using Lipofectamine-2000 (116680-19) according to the manufacturer’s instructions. All cells are tested every three months for mycoplasma contamination in our laboratory.

**Stable transduction**

Lentiviral vectors were produced by seeding 15 x 10^6^ HEK293T cells in a 175 cm2 flask. Twenty-four hours later, the cells were transfected with 37.5 µg pLenti-puro (transfer plasmid containing the construct of interest), 12.5 µg GAG, 6.25 µg REV and 9 µg VSV-G using 130 µg PEI (Polysciences, Eppelheim, Germany). The supernatant containing lentiviral particles were harvested 48 and 72 hours post transfection. Cell debris was removed by centrifugation (5000 rpm, 3 minutes) and the collected supernatant was subsequently filtered using a Corning® 50 mL Tube Top Vacuum Filter System (0.22 µm pore size). Filtered medium containing lentiviral particles was supplemented with 10 µg/mL protamine sulfate.

Cells (250.000 cells/well, 6-well format) were stably transduced by adding 2 ml of the lentiviral vector containing medium. Three days post-infection, the medium was replaced by the cell-line appropriated medium containing 2 µg/ml puromycin.

**Western blot**

Western blot analyses were performed as previously described ^2^. Antibodies were: phospho-MEK1/2 (cell signaling, 9121), total-MEK1/2 (cell signaling, 9122), phospho- ERK1/2 (cell signaling, 4370), total ERK1/2 (cell signaling, 4695), total-CRAF (cell signaling, 9422S), phospho-S338-CRAF (cell signaling, 9427), phospho-S259-CRAF (9421S) HA-TAG (cell signaling, 2367), FLAG (Sigma, F1804), and anti-beta ACTIN (Sigma, A1978). Primary antibodies labeled with near-infrared secondary antibodies (IRDyes 680 RD or 800 CW, LI-COR Biosciences) and were detected and quantified using Odyssey ® Fc Imaging System (LI-COR Biosciences).

**Inhibitors**

Dabrafenib (TAFINLAR) was provided by GlaxoSmithKline. Trametinib (GSK1120212), AZ628, Sorafenib, Vemurafenib, and LY39009120 were obtained from Selleckchem.

**Soft agar colony formation assay**

Cells were seeded in a 6-well plate at a density of 10.000 cells/well suspended in 1.5 ml 0.3% Select agar (Thermo Fisher Scientific, 30-391-023) in medium containing 10% FBS/BCS on top of a solidified bottom layer of 1.5 ml 0.5% % Select agar in DMEM 10% FBS/BCS. Bottom layer and cell seeding in the described top layer are performed according to the previously described protocol^6^. Powder medium (DMEM, Thermo Fisher Scientific, 12-800-017) was used in both top and bottom layers.

Cells were detached using versene and trypsin and cell clusters were excluded using a cell strainer with a pore size of 35 μm (Corning, 352235). Cells were counted using a CASY cell counter (Roche) and counting errors among different cell lines were determined and adjusted for using the CellTiter-Glo® Luminescent Cell Viability Assay (Promega). After seeding, the top layer was allowed to solidify at room temperature for 1 hour. Subsequently, the solubilized agar was topped up with 1 ml of medium (10% FBS/CBS) at the day of seeding. Representative images were made using an EVOS Cell Imaging System (Thermo Fisher Scientific). Colonies were counted manually using a phase contrast microscope (4x magnification). The experiment was terminated when staining the colonies by adding 200 µl of 1 mg/ml Nitro Blue Tetrazolium chloride (438592X, VWR) in PBS. The 6 well plates were photographed 12-16 hours post-staining (using a lightbox and Huawei mate 20 pro phone camera). Relative colony sizes were determined using open CFU software^7^.

**Cell viability assay**

Cell viability was determined using CellTiter-Glo Luminescent assay (Promega, G7570), according to manufacturer's instructions. Briefly, cells were seeded in 96 well plates at 2000 cells/well. Small molecule inhibitors were added 24 hours after seeding. After 72 hours of additional incubation, 100 µl of CellTiter-Glo was added to the wells and luminescent signal was quantified using a Spectramax M3 (Molecular Devices).

**Statistical analysis**

Statistical analysis was performed for the cell viability data. Means of 3 independent experiments (unless indicated differently) were compared by a one-way ANOVA, using the Holm-Šídák post-hoc test.

1 Guan B, Wang T-L, Shih I-M. ARID1A, a factor that promotes formation of SWI/SNF-mediated chromatin remodeling, is a tumor suppressor in gynecologic cancers. *Cancer Res* 2011; **71**: 6718–6727.

2 Noeparast A, Teugels E, Giron P, Verschelden G, De Brakeleer S, Decoster L *et al.* Non-V600 BRAF mutations recurrently found in lung cancer predict sensitivity to the combination of Trametinib and Dabrafenib. *Oncotarget* 2017; **8**: 60094–60108.

3 Rocks N, Estrella C, Paulissen G, Quesada-Calvo F, Gilles C, Gueders MM *et al.* The metalloproteinase ADAM-12 regulates bronchial epithelial cell proliferation and apoptosis. *Cell Prolif* 2008; **41**: 988–1001.

4 Estrella C, Rocks N, Paulissen G, Quesada-Calvo F, Noel A, Vilain E *et al.* Role of A disintegrin and metalloprotease-12 in neutrophil recruitment induced by airway epithelium. *Am J Respir Cell Mol Biol* 2009; **41**: 449–458.

5 Shapira S, Barkan B, Friedman E, Kloog Y, Stein R. The tumor suppressor neurofibromin confers sensitivity to apoptosis by Ras-dependent and Ras-independent pathways. *Cell Death Differ* 2007; **14**: 895–906.

6 Borowicz S, Van Scoyk M, Avasarala S, Karuppusamy Rathinam MK, Tauler J, Bikkavilli RK *et al.* The soft agar colony formation assay. *J Vis Exp* 2014; : e51998–e51998.

7 Geissmann Q. OpenCFU, a new free and open-source software to count cell colonies and other circular objects. *PLoS One* 2013; **8**: e54072.

**supplementary figure legends**

**Fig S1.**

BEAS-2B (**a**), MEF (**b),** and NIH3T3 (**c**) were stably transduced with empty vector, CRAF^WT^, CRAF^P207S^ (BEAS-2B and NIH3T3), CRAF^S259A^, CRAF^P261A^ and BRAF^V600E^ (only BEAS-2B) as indicated. (**a-c)** Total number of colonies per well were counted (representative experiment) after sixteen (**b**) and eighteen (**a, c**) days in culture. The experiments were performed with three biological repeats and were represented as a bar chart (Means ± SEM). (**d**) Representative high magnification images of the NIH3T3 colonies at day 25. (**e**) Representative high magnification images of the BEAS-2B colonies at day 25. (**f**) Representative whole well image of the nitro blue tetrazolium chloride stained BEAS2B colonies after 28 days in culture (zoom-in the digital image for clarity).

**Fig S2.**

BEAS-2B cells were transiently co-transfected with the indicated CRAF expression vectors together with the BRAF^WT^ expression vector. Forty-eight hours post-transfection cells were treated for 2h with DMSO, Dabrafenib (1 µM) or AZ628 (1 µM) or LY3009120 (1 µM), then lysed and subjected to western blot analysis for the indicated proteins.

**Fig S3.**

BEAS-2B cells were transiently co-transfected with the indicated CRAF expression vectors together with the BRAF^WT^ expression vector. Forty-eight hours post-transfection cells were treated for 2h with DMSO, or LY3009120 (1 µM) or/and Trametinib (25 nM), then lysed and subjected to western blot analysis for the indicated proteins.
