# Supplementary figures and images for: CRAF mutations in lung cancer can be oncogenic and predict sensitivity to combined type II RAF and MEK inhibition
Source: Oncogene. 2019 Jul 8;38(31):5933–41. doi: 10.1038/s41388-019-0866-7 (PMC6756226; doi:10.1038/s41388-019-0866-7)

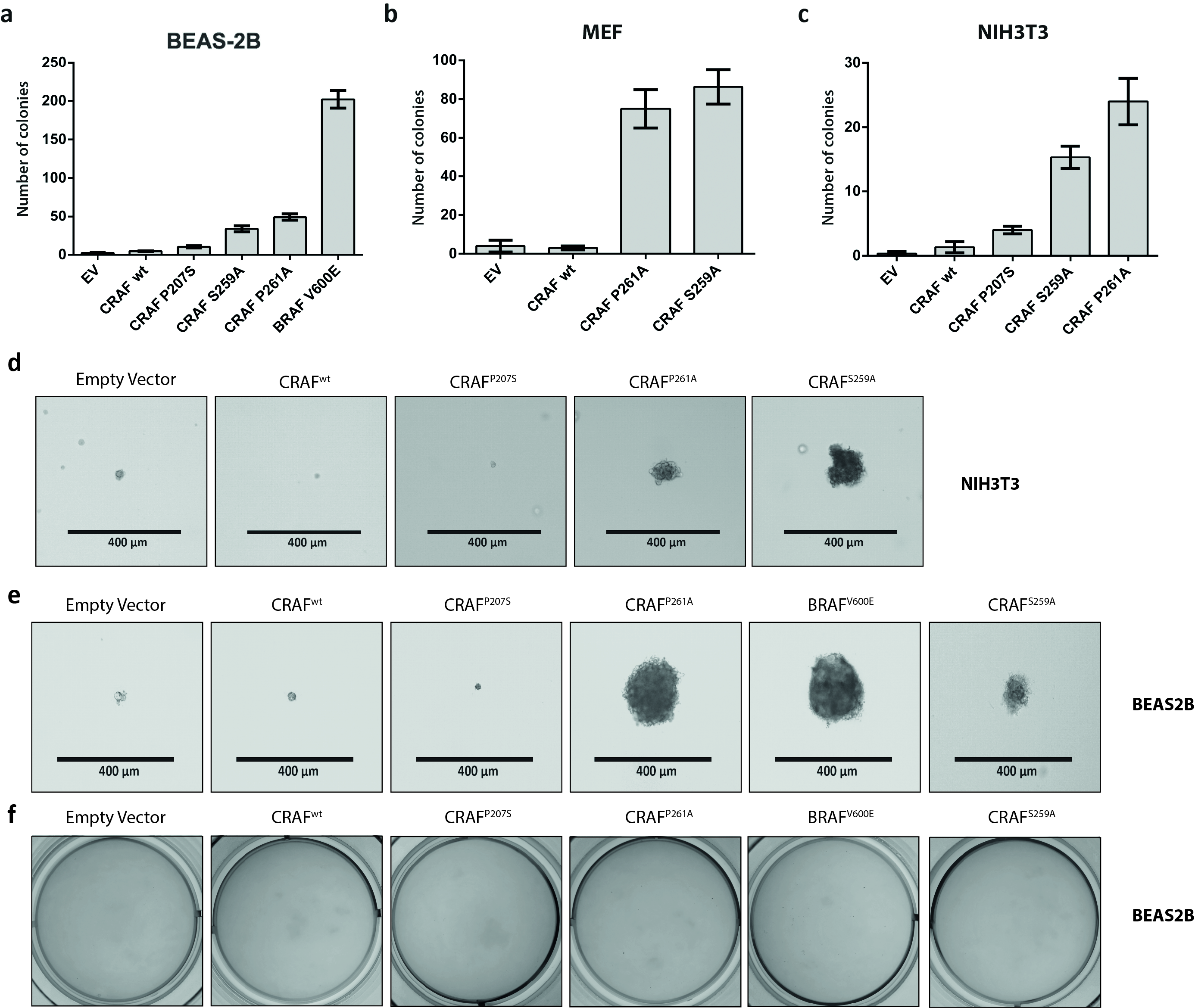

Supplement: Supplementary file 2 — S1 [file 41388_2019_866_MOESM2_ESM.tif]

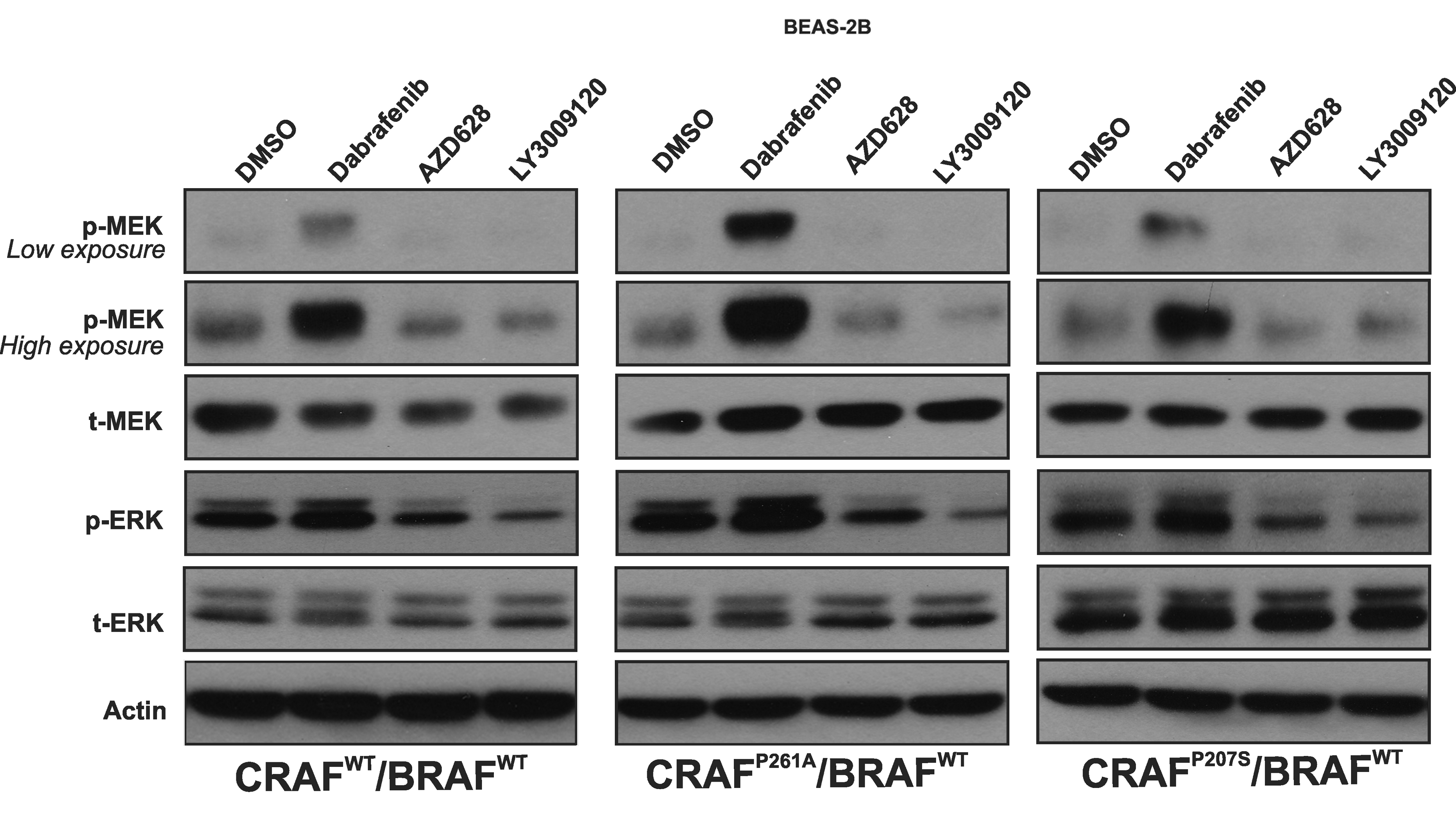

Supplement: Supplementary file 3 — S2 [file 41388_2019_866_MOESM3_ESM.tif]

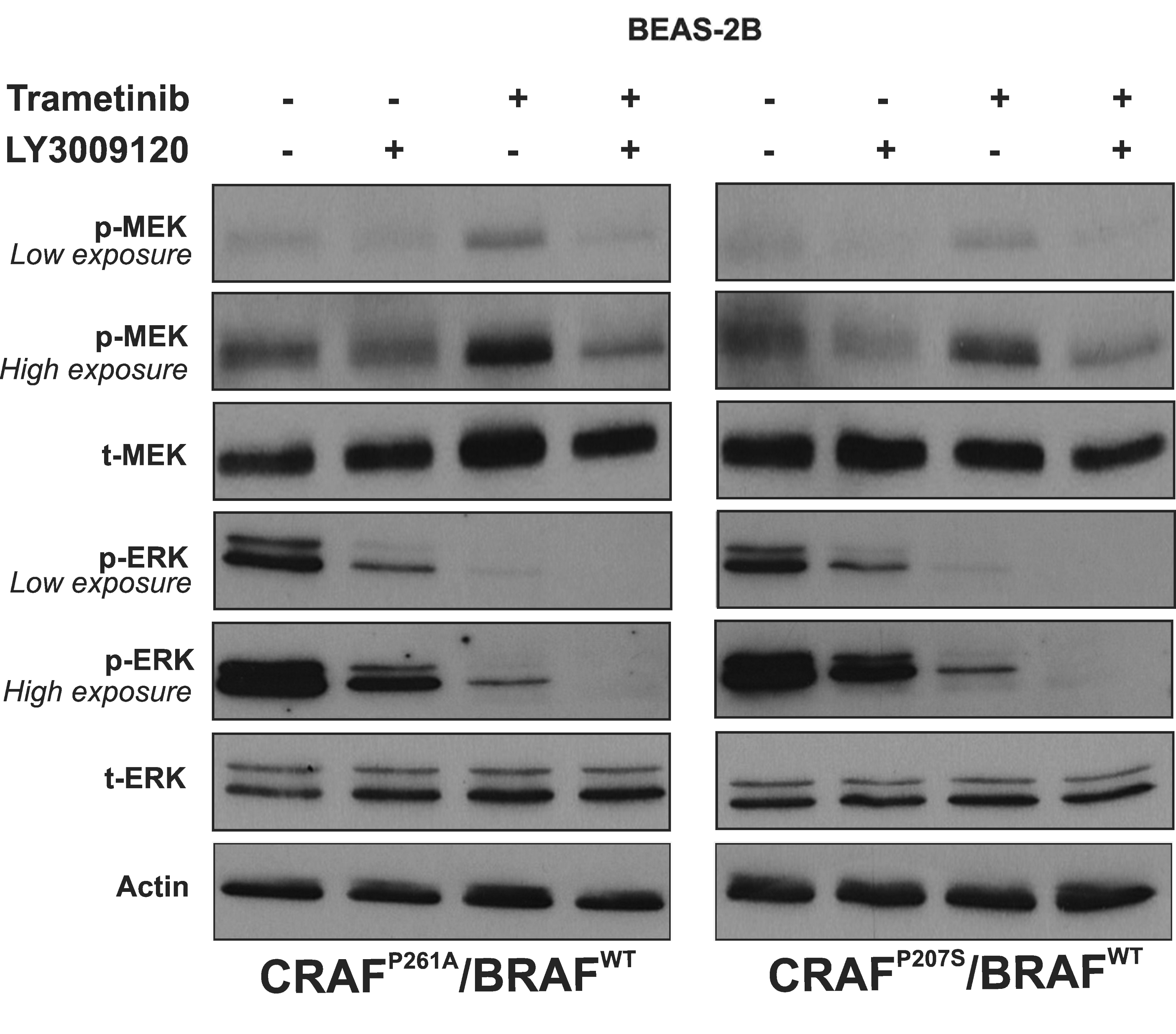

Supplement: Supplementary file 4 — S3 [file 41388_2019_866_MOESM4_ESM.tif]
